# Supplementary material for: Virtual Connectomic Datasets in Alzheimer’s Disease and Aging Using Whole-Brain Network Dynamics Modelling
Source: eNeuro. 2021 Jul 3;8(4):ENEURO.0475-20.2021. doi: 10.1523/ENEURO.0475-20.2021 (PMC8260273; doi:10.1523/ENEURO.0475-20.2021)
Supplement: Extended Data Table 1-1 — provides a pseudo-code for the linear SC-to-FC completion procedure (see Materials and Methods for all details). Linear SC-to-FC completions for the DTI-only subjects in the considered ADNI dataset and the healthy ageing dataset can be downloaded as part of Extended Data 1 FC_SLM. Download Table 1-1, DOCX file. [file enu-eN-MNT-0475-20-s11.docx]

***Extended Data Table 1-1. Pseudo-code for linear SC-to-FC completion***

**algorithm** linear SC-to-FC completion **is**

**external input:** empirical SC (SC_emp_)

**output:** linear virtual FC (FC_SLM_)

**fixed parameters:** noise level ($\sigma$), guess for optimal G (G*_ref_)

**begin**

1. Evaluate the covariance matrix C from SC_emp_ based on SLM theory for different range of G
2. Choose G* as a G which rise to maximum correlation between FC_emp_ and FC_SLM_ for each subject
3. Choose G*_ref_ as the median of G* for all subjects
4. Re-evaluate the covariance Matrix C for G*_ref_

**return** FC_SLM_ = C

**end**

***Extended Data Table 2-1. Pseudo-code for linear FC-to-SC completion***

**algorithm** linear FC-to-SC completion **is**

**external input:** empirical FC (FC_emp_)

**output:** linear virtual SC (SC_SLM_)

**fixed parameters:** noise level ($\sigma$), guess for optimal G (G*_ref_)

**begin**

1. Evaluate the inverse matrix C^-1^ from FC_emp_
2. Build a matrix S proportional to C^-1^ according to SLM theory and drop its diagonal

**return** SC_SLM_ = S

**end**

**Extended Data Table 3-1. Discriminating control and patient subjects in the ADNI subset with only SC connectomes.**

| *Tested on* | SC_emp_ | FC_MFM_ | SC_biMFM_ |
| --- | --- | --- | --- |
| *Trained on* |  |  |  |
| SC_emp_ | 0.69 *[0.61 < AUC < 0.74]* | n.s. | 0.55 *[0.51 < AUC < 0.60]* |
| FC_MFM_ | 0.54 *[0.50 < AUC < 0.59]* | 0.64 *[0.57 < AUC < 0.69]* | 0.62 *[0.54 < AUC < 0.68]* |
| SC_biMFM_ | 0.56 *[0.51 < AUC < 0.62]* | 0.62 *[0.53 < AUC < 0.68]* | 0.59 *[0.52 < AUC < 0.64]* |

*Indicated values are median and 5% and 95% percentiles over crossvalidation replicas of the indicated classification.*

**Extended Data Table 3-2. Discriminating control and patient subjects in the ADNI subset with only FC connectomes.**

| *Tested on* | FC_emp_ | SC_MFM_ | FC_biMFM_ |
| --- | --- | --- | --- |
| *Trained on* |  |  |  |
| FC_emp_ | 0.75 *[0.70 < AUC < 0.79]* | 0.71 *[0.65 < AUC < 0.79]* | 0.65 *[0.58 < AUC < 0.71]* |
| SC_MFM_ | 0.69 *[0.61 < AUC < 0.75]* | 0.73 *[0.67 < AUC < 0.78]* | 0.55 *[0.50 < AUC < 0.61]* |
| FC_biMFM_ | 0.70 *[0.65 < AUC < 0.76]* | n.s. | n.s. |

*Indicated values are median and 5% and 95% percentiles over crossvalidation replicas of the indicated classification.*

**Extended Data Table 5-1. Inter-subject distances for empirical – bivirtual pairs.**

| Type of completion | | Inter-distance correlation | |
| --- | --- | --- | --- |
|  |  | ADNI | Healthy aging |
| SCemp to SCemp vs | SCbiSLM to SCbiSLM | 0.39*** [0.37, 0.41] | 0.81*** [0.79, 0.83] |
|  | SCbiMFM to SCbiMFM | 0.39*** [0.36, 0.43] | 0.53*** [0.48, 0.57] |
| FCemp to FCemp vs | FCbiSLM to FCbiSLM | 0.06*** [0.5, 0.8] | 0.55*** [0.50, 0.58] |
|  | FCbiMFM to FCbiMFM | 0.43*** [0.42, 0.44] | 0.40*** [0.36, 0.44] |

*Indicated values are median and 5% and 95% percentiles over bootstrap with replacement replicas of correlation computation*
